# Assessment of fluid responsiveness in spontaneously breathing critically ill patients

**DOI:** 10.1186/cc14688

**Published:** 2015-09-28

**Authors:** Renato CF Chaves, Murillo SC Assunção

**Affiliations:** 1 Department of Intensive Care Medicine, Hospital Santa Lúcia, Brasília, DF, Brazil; 2 Department of Intensive Care Medicine, Hospital Israelita Albert Einstein, São Paulo, SP, Brazil

## Introduction

One of the main challenges in critical patient management is to assess the blood volume and determine which patients will benefit from volume expansion and which patients will benefit from support with vasopressor and/or inotropic drugs. It is known that 40-72 % of critical patients respond to volume expansion with increased stroke volume or cardiac index.

## Objective

To search the literature for methods assessing fluid responsiveness in spontaneously breathing critically ill patients.

## Methods

The present study is a systematic literature review. We searched randomized clinical trials through a blind search performed by two independent authors in any language in the National Library of Medicine from 2009 to 2014.

## Results

We selected three articles for full review and analysis, totaling 116 patients. The results are shown in Table 1.

**Table 1:** Accuracy of hemodynamic parameters for predicting fluid responsiveness.

	**Préau et al., 2010 **[4]**(*n *= 34) **			**Préau et al., 2012**[2]**(*n *= 23)**				**Hong et al., 2014 **[3]**(*n *= 59)**	
	
Parameter	ΔSV-PLR	ΔPP-PLR	ΔVF-PLR	ΔPP	ΔPP-dim	ΔVF	ΔVF-dim	ΔPP-TB	ΔPP-FB
Threshold value (%)	10	9	8	10	12	10	12	NS	13.7
Sensitivity (%)	86	79	86	60	90	60	90	NS	89.7
Specificity (%)	90	85	80	100	100	100	100	NS	86.7
ROC curve area	0.94	0.86	0.93	0.71	0.95	0.74	0.95	0.618	0.910
*p *value	<0.001	<0.01	<0.001	0.02	<0.01	0.02	<0.01	0.112	<0.0001
Responders	14	14	14	10	10	10	10	29	29

*Dim *deep inspiration maneuver-induced change, *FB *forced inspiratory breathing, *NS *non specified, *PLR *passive leg raising induced change, *ΔPP *pulse pressure, *ΔSV *stroke volume, *TB *tidal breathing, *ΔVF *peak velocity of femoral artery flow

## Conclusion

This systematic review supports the beneficial effects of adopting maneuvers that amplify the hemodynamic changes, increasing the accuracy of methods to predict fluid responsiveness in spontaneously breathing critically ill patients.
